# Digital body mapping of pain quality and distribution in athletes with longstanding groin pain

**DOI:** 10.1038/s41598-022-13847-1

**Published:** 2022-06-13

**Authors:** Andreas Serner, Gilles Reboul, Olivier Lichau, Adam Weir, Willem Heijboer, Zarko Vuckovic, Shellie Ann Boudreau

**Affiliations:** 1 Aspetar Orthopaedic and Sports Medicine Hospital, Sport City Street, PO Box 29222, Doha, Qatar; 2 Present Address: Fédération Internationale de Football Association, FIFA-Strasse 20, P.O. Box 8044, Zurich, Switzerland; 3 Hernia Center, Clinique du Sport, Bordeaux-Mérignac, 4 Rue Georges Negrevergne, 33700 Mérignac, France; 4 Hôpital Pellegrin, Centre Hospitalier Universitaire, Place Amélie Raba Léon, 33000 Bordeaux, France; 5 Department of Orthopaedics and Sports Medicine, Erasmus MC University Medical Centre, Rotterdam, The Netherlands; 6 Amsterdam University Medical Centers, Academic Medical Center Amsterdam, Amsterdam Movement Sciences, Academic Center for Evidence Based Medicine, Amsterdam IOC Center, ACHSS, Amsterdam, The Netherlands; 7 Center for Neuroplasticity and Pain (CNAP), Department of Health Science and Technology, Aalborg University, Aalborg, Denmark

**Keywords:** Pain, Physical examination, Musculoskeletal system

## Abstract

Groin pain is common in athletes, but remains a challenge to diagnose. Self-reported pain quality distribution may facilitate differential diagnoses. We included 167 athletes with groin pain (≥ 4 weeks). All athletes received a standardized clinical examination. Athletes could choose multiple quality descriptors and intensity, and drew these on a digital body map. Overlay images were created to assess distribution and area visually. Intensity, duration, and qualities were compared between each clinical entity and multiple entities. Top three quality descriptors were electric (22%), pain (19%), and dull/aching (15%). There were no differences in the frequencies of quality descriptors (p = 0.893) between clinical entities. Areas of the mapped qualities were similar between the single clinical entities (χ^2^(3) = 0.143, p = 0.986) and independent of symptom duration (ρ = 0.004, p = 0.958). Despite a considerable overlap, the mapped pain qualities’ distributions appear to differ visually between single clinical entities and align with the defined clinical entities of adductor-related, inguinal-related, and pubic-related groin. In iliopsoas-related groin pain, pain extended more medially. The overlap between the drawn areas underscores a challenge in differentiating groin pain classifications based only on self-reported pain. The prevalence of pain quality descriptors varied and individually do not associate with one particular clinical entity of groin pain.

## Introduction

Groin pain in athletes is often considered an enigma within sports medicine. As a result, diagnostic terminology and definitions have been confusing, with different terms used to describe similar symptoms or the same terms used for different types of symptoms^1,2^. The groin region is in itself poorly defined without clear anatomical borders. According to the Medical Subject Headings thesaurus, the groin is defined as “the external junctural region between the lower part of the abdomen and the thigh”^3^. There is no agreement on the etiology or origin of pain in athletes with longstanding groin pain. In 2015, international groin experts published the “Doha agreement meeting on terminology and definitions in groin pain in athletes”^2^. This agreement outlines a classification system based on a clinical examination with three major subheadings: (1) Four defined clinical entities of groin pain: adductor-related, iliopsoas-related, inguinal-related, and pubic-related groin pain, (2) Hip-related groin pain, and (3) Other causes of groin pain. The categorizations of the defined clinical entities rely on clinical pain provocation tests and the history of the pain location. However, a clear description of what is meant by pain is lacking. Pain is an abstract and multidimensional experience that can be reported by patients differently.

Prior research of self-reported pain locations on the body using digital body maps shows patients can communicate, by way of pain drawings, remarkable detail of pain on and around the knee^4,5^. An analysis of these detailed drawings revealed distinct location patterns or distribution of pain in patients with a common knee pain syndrome known as patellofemoral pain^6^. These distinct patterns provide a starting point for exploring underlying structures or driving mechanisms of pain^6^. To date, no studies have explored the detailed location of self-reported pain using digital body mapping combined with the intensity and quality of pain within a specific clinical condition or diagnosis. The pain quality descriptors, for example, provide clinical insight into the type of pain and can facilitate clinical reasoning for differentiating the type (e.g. neuropathic or nociceptive pain) or drivers of pain.

We aimed to explore self-reported pain locations and quality of pain in athletes with longstanding groin pain, classified according to the Doha agreement meeting classification system. Pain location and qualities of pain were assessed for the area (total pixels), distribution, intensity, laterality (bilateral vs. unilateral), and symmetry in those with bilateral pain.

## Methods

### Participants

Adult athletes with groin pain from any sport at any level were consecutively recruited from two clinics of a general surgeon (GR) specialized in groin pain in athletes. The clinics are in Bordeaux and Paris, France, and the population includes athletes from all over France. Inclusion criteria were: Male and female elite and recreational athletes (usually performing sports ≥ 1×/week) with sports-related groin pain ≥ 4 weeks duration, age: 18 + years, and a clinical classification of one or more of the defined clinical entities of groin pain (adductor- inguinal-, iliopsoas- and/or pubic-related groin pain). Exclusion criteria were: A classification of hip-related groin pain (clinical suspicion that the hip joint is the source of groin pain, through history and/or clinical examination) or other causes of groin pain (clinical suspicion that the groin pain cannot be classified into one of the described clinical entities, with or without additional investigations, e.g. spinal pathology, prostatitis, urinary tract infections, or other medical conditions considered to potentially influence the pain presentation), and prior surgery in the area of the current clinical entity of groin pain or any surgery in the groin region within the previous year.

### Ethics

Ethical approval was granted by the ethics committee of Clinique du sport de Bordeaux-Merignac, France (#02-2020.1). Athletes were included in this study after giving informed consent according to the Declaration of Helsinki. Study participants could withdraw at any time point without reporting a reason and without any consequences.

### Patient information

Demographic data (age, height, weight, type and level of sport/competition, and duration of groin pain) were recorded.

### Acquisition of pain drawings

Athletes drew their area of pain on a high-resolution and detailed digital-body chart using the Navigate Pain web application (Aglance Solutions ApS, Denmark) and a computer tablet (iPad Air 1). The use of digital collection methods for obtaining pain drawings is a valid approach and comparable to conventional paper methods^5^. Digital pain drawings may be a more precise method for mapping pain. The body charts are approximately 3–4 times larger on the computer tablet than shown on paper in conventional questionnaires^7^. The body chart clearly shows the anatomical detail around the groin area and provides a considerable amount of space for drawing. The application also includes zooming and manoeuvring to improve accuracy of drawing. Participants used a stylus pen, making the drawing precise to the stylus’s tip size, approximately 1.5 mm. As inguinal-related groin pain may involve nerve symptoms extending to the scrotum^8,9^, an additional body chart detailing the male genitals was developed and given as an option for all participants. Before the clinical examination, participants created a pain drawing according to the following instructions, which were given verbally by the surgeon:

1. Draw one or more areas of current groin pain in the groin area on the image.
2. Choose different colors according to the pain quality and intensity.
3. Draw as accurately as possible and to the best of your ability.
4. Shade pain areas entirely and avoid using circle outlines or cross-marks for pain areas.

**Figure 1:**
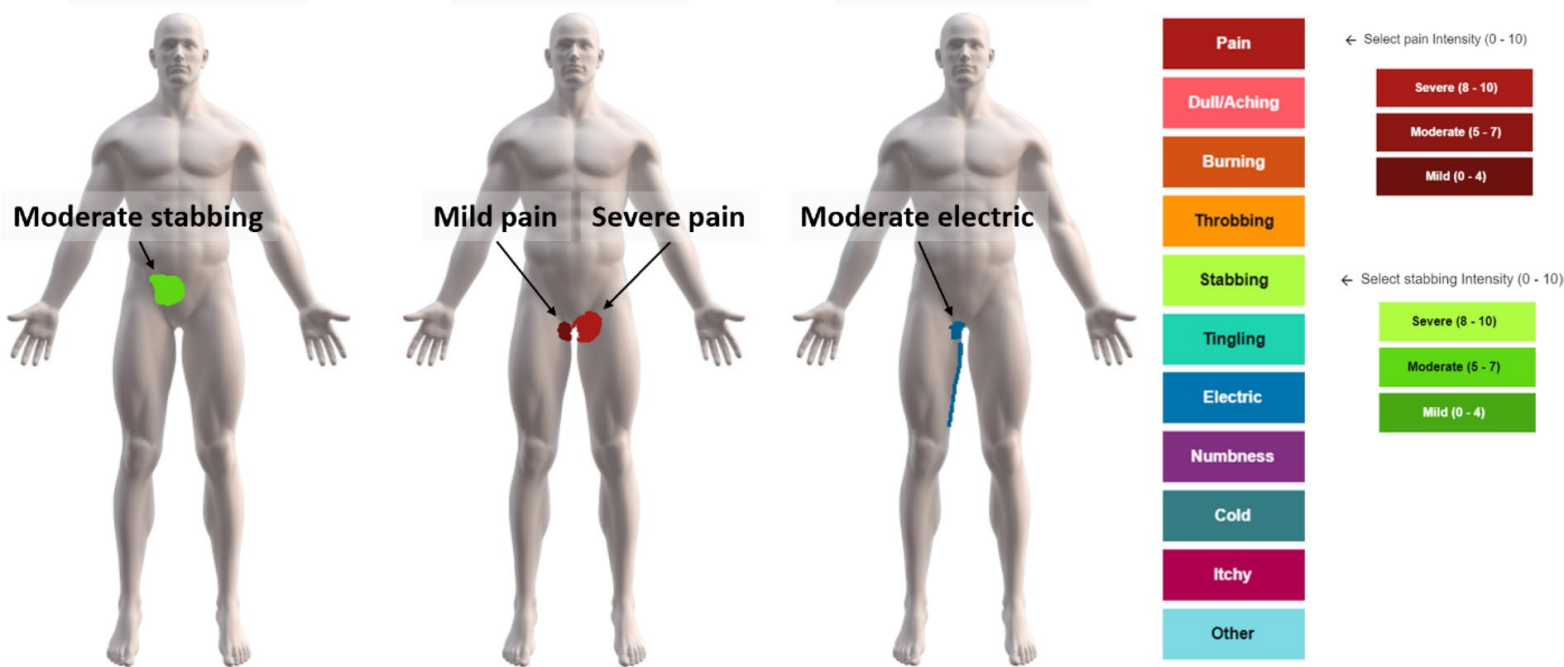
Examples of the anatomically detailed pain charts and individual pain drawings showing different pain quality descriptors, pain intensities, and pain areas. Green indicates the pain quality “stabbing” (2330 pixels), red indicates “pain” (1800 pixels), and blue “electric” (1485 pixels). Colour shading from dark to light indicates pain intensity (mild, moderate, severe).

All participants could select between nine different pain qualities (dull/aching, burning, throbbing, stabbing, tingling, electric, numbness, cold, and itchy) and “pain” from a drop down list. Patients could choose “pain” if they could not determine the pain quality or preferred to use this term. It was possible to use multiple pain descriptors in a single pain drawing. Immediately after selecting a pain quality, the application prompts the user to select a mild, moderate, or severe intensity rating, corresponding to a numerical pain rating scale of 0–4/10, 5–7/10, or 8–10/10, respectively—examples in Fig. 1.

### Clinical diagnosis

A clinical examination was performed, blinded to the pain drawings, by the same general surgeon (GR) for all patients. The surgeon was specialized in groin pain in athletes with 25 years of experience. Injury history and pain provocation tests were used to classify patients’ groin pain according to the defined clinical entities of the Doha agreement meeting classification system^2^:

1. *Adductor-related groin pain*: Adductor tenderness and pain on resisted adduction testing.
2. *Iliopsoas-related groin pain*: Iliopsoas tenderness, and more likely if there is pain on resisted hip flexion and/or pain on stretching the hip flexors.
3. *Inguinal-related groin pain*: Pain location in the inguinal canal region and tenderness of the inguinal canal. No palpable inguinal hernia is present. More likely if the pain is aggravated with resistance testing of the abdominal muscles OR on Valsalva/cough/sneeze.
4. *Pubic-related groin pain*: Local tenderness of the pubic symphysis and the immediately adjacent bone.

Athletes can be classified with a single entity or multiple entities of groin pain.

### Assessment of pain distribution

Pain map overlays enable a visual exploration of common pain distribution associated with a particular clinical grouping. In this study, pain map overlays combine the pain descriptors into one map for each single entity of groin pain, and one, two, and three clinical entities of groin pain. The pain map overlays utilize the original pain drawings, but do not necessarily reflect the original drawing colour. All pain map overlays were created in the Navigate Pain web application using the overlay functionality with a Jet colour-scheme from dark blue to dark red to show the relative frequency for each grouping. The colour-scheme scale reflects the maximum overlap for each clinical grouping. Before creating the pain map overlays, common regions of pain across single entities of groin pain and between two or three clinical entities of groin pain are made clear by flipping unilateral left-sided pain drawings to the right. Similarly, bilateral pain drawings with the largest drawn area are flipped to the right side to create a more clear image where all drawings are on the same side. Lastly, each overlay threshold is a minimum of one overlapping area, with areas drawn by only one individual excluded in the summary figures. Due to the low number of females included, pain map overlays were only included for the males. In addition to the summary figures, we traced the border of the total pain area and the most common locations (≈ 75%) for the overlay images of each of the four entities with a stylus pen to visualize the overlap between pain areas between the entities.

### Assessments of pain area

For each pain drawing, the area, expressed as pixels, is associated with a self-reported pain descriptor and an intensity. Pain descriptors, intensities, and areas were automatically extracted. These areas were considered separate contributions in the assessment of pain descriptor area and frequency within and between the clinical entities of groin pain for athletes reporting one or more pain descriptors. Analyses of the overall pain area excluded any overlap between different pain descriptors drawn in the same areas in a pain drawing. For example, a pain drawing with moderate “pain” and a mild electric sensation drawn in the same area would only contribute to the overall pain area calculation once, whereas both pain descriptors were utilized for frequency assessments.

### Assessments of pain laterality and symmetry

Each pain drawing was assessed for laterality and rated as having either unilateral or bilateral pain by an assessor not involved in the clinical care (SB). Bilateral pain was rated when the area of pain crossed the midline of the body chart. All bilateral pain drawings were rated as symmetric, borderline symmetric, or asymmetric according to previous work^4^ and confirmed by a clinical assessor (AS). Symmetric pain appears as a mirror image between the left and right side of the body, borderline symmetric pain covers the same areas on the left and right side of the body to a moderate extent, and asymmetric pain covers the same areas to a low extent, if at all. Prior research also shows that the clinical assessment of symmetry is more strict than an automatic assessment^4^.

### Statistical analysis

Overall pain area was not normally distributed for adductor-, inguinal-, and iliopsoas-related groin pain, one and two clinical entity groupings, and unilateral and bilateral groupings (Shapiro–Wilk’s test, p < 0.05). We used a Kruskal–Wallis H-test when comparing pain area and pain intensity for these variables. We also used the Kruskal–Wallis H-test to determine if the pain area differs between pain descriptors within each of the four clinical entities. Box plots of the distributions were assessed visually, and differences in distribution or medians reported where appropriate. Results are reported for mean ranks unless specified otherwise. To assess the relationship between duration of groin pain and the pain area for all athletes and athletes with a single entity of groin pain, we used Spearmans rank correlation. To assess whether there was a difference in pain duration between the number of clinical entities, we used the Kruskall Wallis-H-test. We used the Mann–Whitney U-test to determine differences in pain area between unilateral and bilateral clinical entities. We used the chi-square goodness-of-fit test to assess the proportion or relative frequency of pain descriptor intensity for the whole cohort and to determine differences in the proportion of pain descriptors between adductor-, inguinal-, iliopsoas-, and pubic-related groin pain.

All analyses were carried out in Statistical Package for Social Sciences (SPSS; version 27, IBM). We used Bonferroni corrections for multiple comparisons, and present only adjusted p-values in the results. Missing data were excluded from analyses.

### Sample size

As this is the first study of its kind, and because groin pain comprises different entities and combinations of entities of groin pain, any sample size estimate would come with a high level of uncertainty. Therefore we chose an exploratory approach using a convenience sample with an inclusion period of 4 months (Nov 2019–Feb 2020). We accepted the risk of having inadequate numbers for the least frequent entities and female athletes, initially estimated to comprise less than 5% of all patients seen in the two clinics.

### Ethical permissions

Ethical approval was granted by the ethics committee of Clinique du sport de Bordeaux-Merignac, France (#02-2020.1).

## Results

### Participant demographics

During the inclusion period, 249 potentially eligible patients completed pain drawings. Of these, 65 were excluded due to a groin surgery within the previous year, 11 were excluded due to a prior surgery in the current area of groin pain, 5 were excluded due to a classification of hip-related groin pain, and 1 was excluded due to a diagnosis classified as “other causes of groin pain.” Patient demographics of the 167 included patients is presented in Table 1.

**Table 1:** Patient demographics (n = 167).

Sex	Male: 152 (94%), Female: 15 (6%)
Age (years)	33 ± 10
BMI	24.4 ± 3.7
Sport	Football: 68 (41%)
	Running: 35 (21%)
	Rugby: 20 (12%)
	Other*: 44 (26%)
Level**	Professional : 20 (12%)
	Semi-professional: 24 (14%)
	Amateur: 115 (69%)
Pain duration (months)***	12 [6–24 ]

Data presented as mean ± SD, median [interquartile range] or number (percentage). * Other sports included more than 20 different sports.** 8 athletes (5%) did not report their level of sport. *** 22 athletes (13%) had missing data on pain duration. Ten patients had a pain duration less than 3 months.

### Clinical diagnoses

Of the athletes included, 88 were classified with one clinical entity of groin pain, whereas the remaining 79 had multiple entities (64 had two and 15 had three), as summarized in detail in Table 2. Overall, adductor-related groin pain was most common (N = 104), followed by inguinal-related (N = 75), iliopsoas-related (N = 41), and pubic-related (N = 41) groin pain.

**Table 2:** Summary of diagnosed clinical entities, gender proportion, prevalence of bilateral clinical entities, bilateral pain drawings (pain drawed across the midline of the body), and symmetric pattern of pain (drawing covers the same areas on each side to a high extent).

Clinical entity	Total	Male/female	Bilateral clinical entities	Bilateral pain drawing	Symmetric pain drawing
**One clinical entity**					
Adductor-related	39	36/3	11	17	10
Inguinal-related	24	22/2	10	6	3
Iliopsoas-related	18	16/2	3	3	0
Pubic-related	7	6/1	-	3	3
**Two clinical entities**					
Adductor- & inguinal-related	25	24/1	16	10	2
Adductor- & pubic-related	18	17/1	10	13	9
Adductor- & iliopsoas-related	7	6/1	3	3	1
Inguinal- & iliopsoas-related	9	9/0	5	3	0
Inguinal- & pubic-related	3	3/0	2	2	1
Iliopsoas- & pubic-related	2	0/2	2	2	1
**Three clinical entities**					
Adductor-, inguinal-, & pubic-related	10	9/1	7	6	4
Adductor-, inguinal-, & iliopsoas-related	4	3/1	3	3	0
Adductor-, iliopsoas-, & pubic-related	1	1/0	0	0	0
**Total**	**167**	**152/15**	**72**	**71**	**34**

Reported as number of patients.

There was a slightly higher proportion of unilateral (N = 95, 57%) than bilateral (N = 72, 43%) clinical entities for the whole cohort (Table 2). The proportion of athletes with bilateral clinical entities was 27%, 59%, and 66% for athletes diagnosed with one, two, and three entities, respectively. Of the athletes with confirmed bilateral clinical entities (N = 72), 38% (N = 27) showed symmetric pain patterns, and 21% (N = 15) borderline symmetry. The groin pain drawings showed cases with pain reported only unilaterally despite bilateral clinical entities (21 out of 72) and vice versa, bilaterally reported pain in cases with unilateral clinical entities (20 out of 95). Based on the pain drawings, 43% (N = 71) of the athletes experienced pain bilaterally (Table 2). Of athletes with bilateral pain drawings, symmetric pain was seen in 48% (N = 34), and 30% (N = 21) showed borderline symmetry.

### Pain distribution

**Figure 2:**
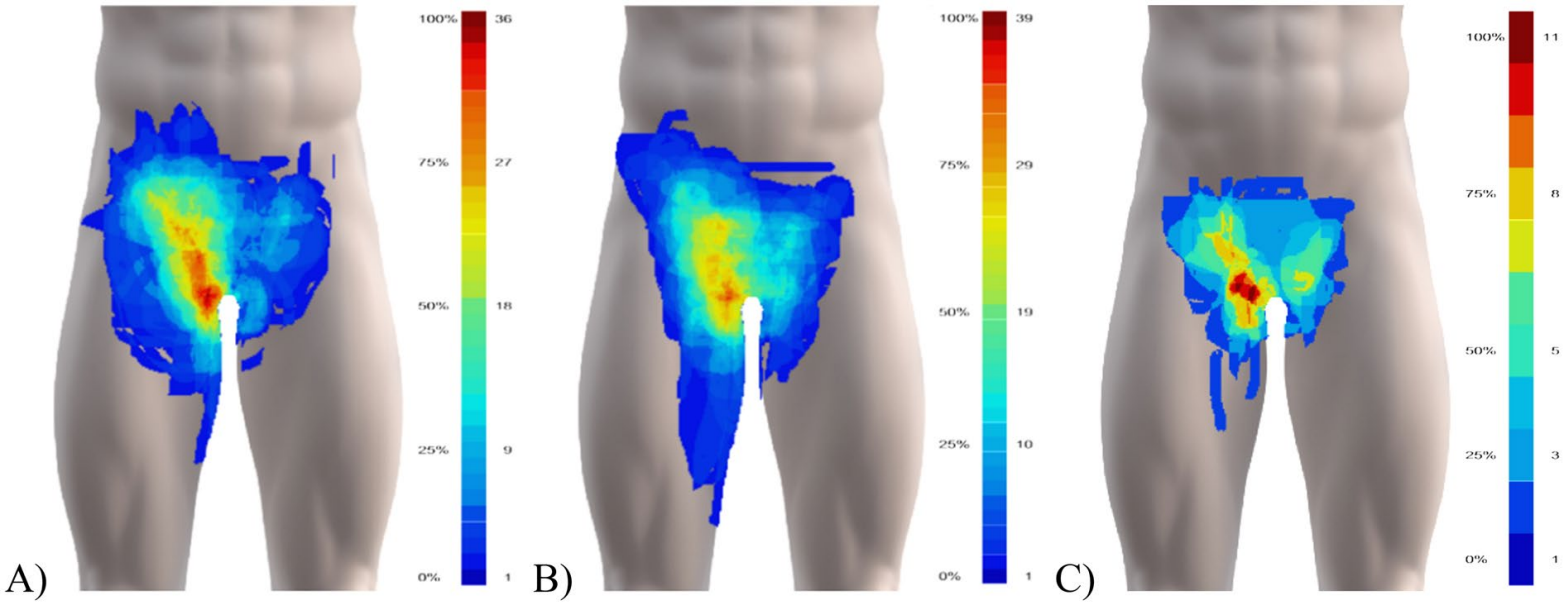
Pain map overlays from patients with (**A**) one (N = 88), (**B**) two (N = 64), and (**C**) three (N = 15) clinical entities of groin pain showing the overall spread and common regions. The colour-scheme range reflects the maximum overlap and the minimum one overlapping area, shown in percent and the absolute number of drawings. Areas drawn by only one individual are not shown in the summary figures. Before overlaying, unilateral left-sided pain drawings were flipped right, and bilateral pain drawings flipped, so the largest drawn area is on the right side.

**Figure 3:**
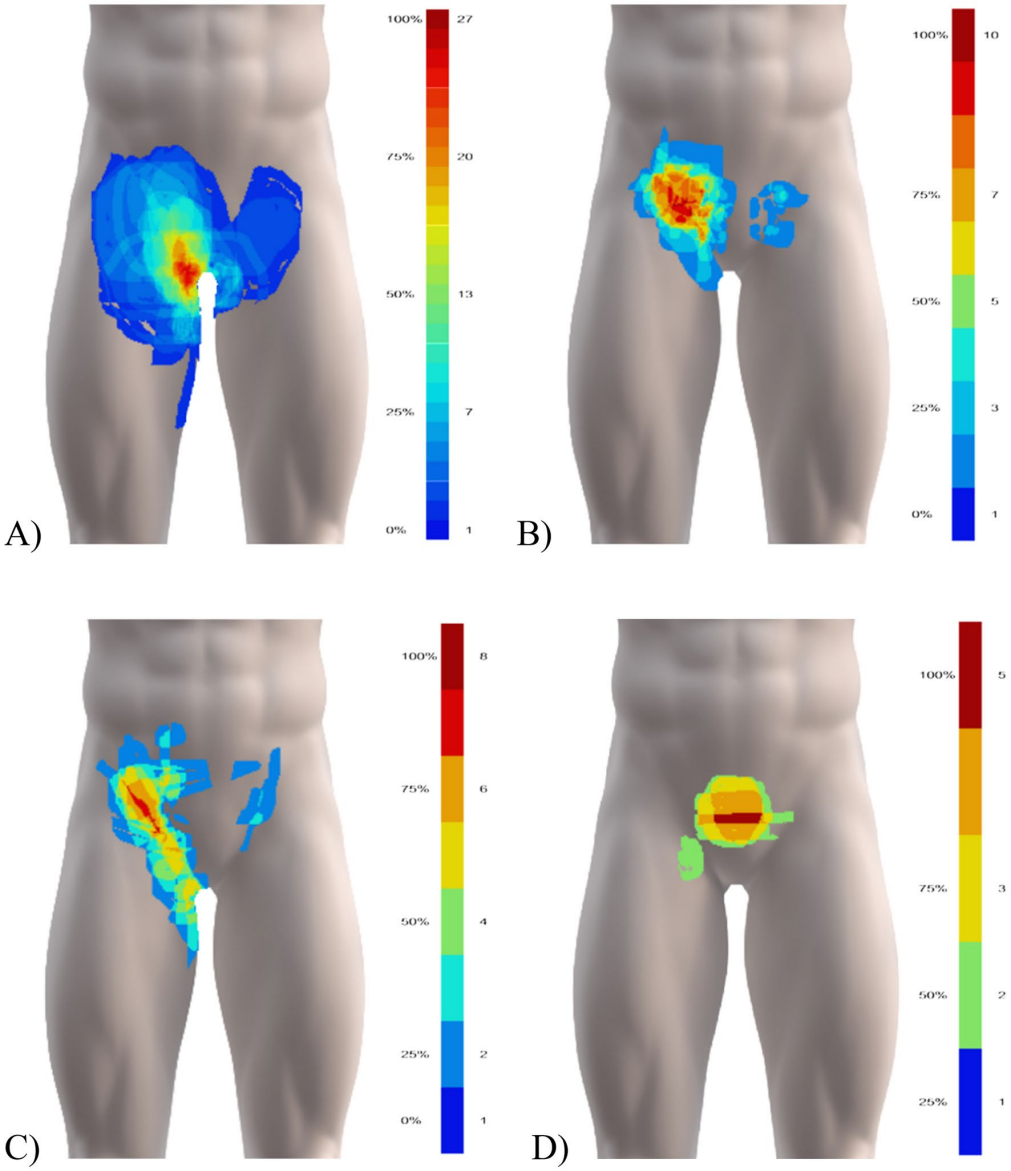
Pain map overlays of: (**A**) adductor- (N = 39), (**B**) inguinal- (N = 24), (**C**) iliopsoas- (N = 18), and (**D**) pubic-related (N = 7) groin pain showing common regions for each clinical entity. The colour-scheme range reflects the maximum overlap and the minimum one overlapping area, shown in percent and the absolute number of drawings. Areas drawn by only one individual are not shown in the summary figures. Before overlaying, unilateral left-sided pain drawings were flipped right, and bilateral pain drawings flipped, so the largest drawn area is on the right side.

**Figure 4:**
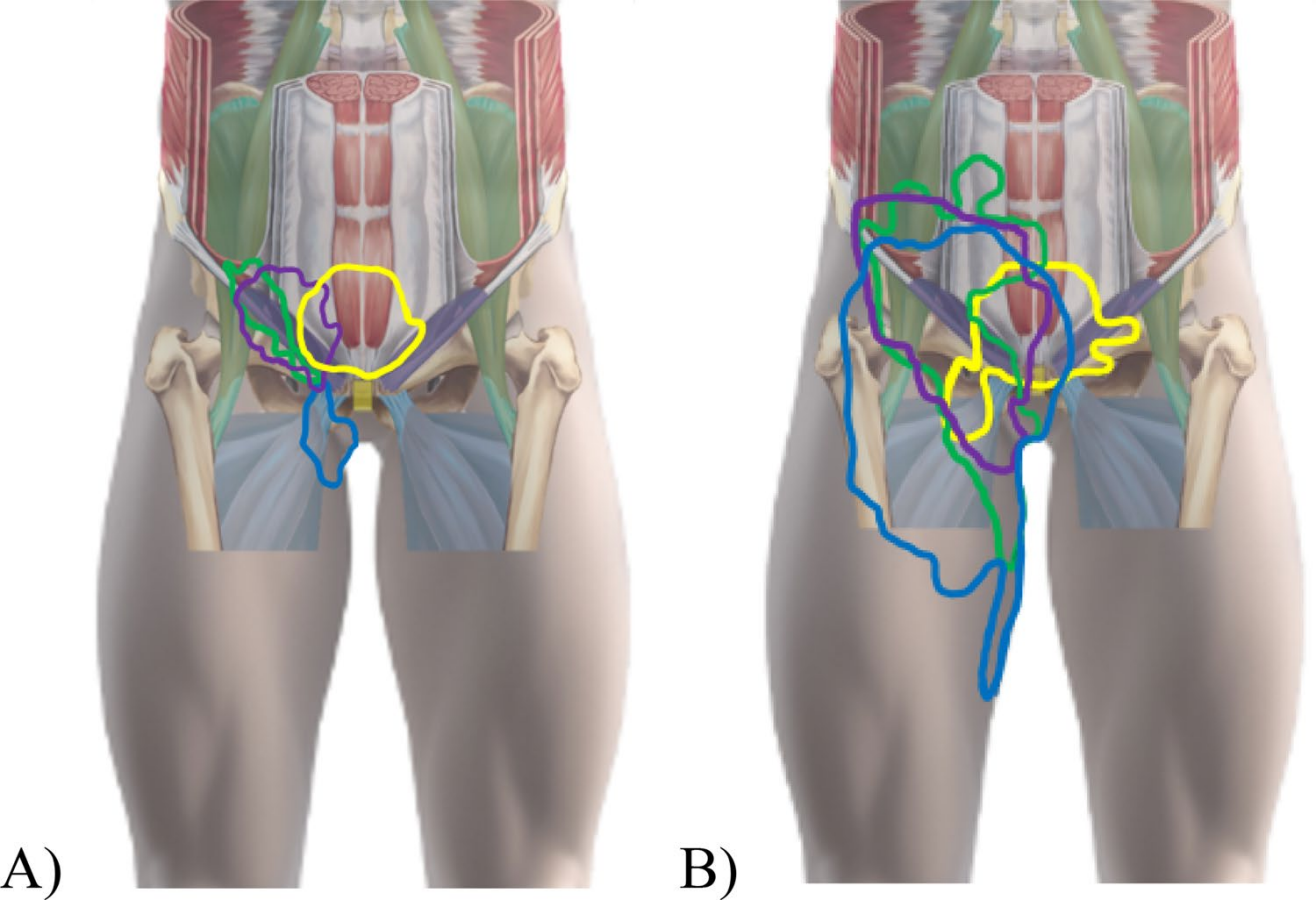
Distributions of pain incorporating the Doha agreement illustration of the four defined clinical entities, using the outlines from adductor-related (blue), inguinal-related (purple), iliopsoas-related (green), and pubic-related (yellow) groin pain from the pain drawing overlay images. All data are flipped to the right side to show common locations. (**A**) Illustrates the area where the majority (≈75%) of athletes reported their pain, whereas (**B**) illustrates the area where a minimum of two athletes reported their pain.

The pain map overlays showed a pain distribution covering most of the groin region following a triangular pattern; extending from the anterior superior iliac spine medially to the linea alba, and distally towards the medial femoral condyle (Fig. 2). No patients drew directly on the scrotum area, so pain drawings are presented without the genitalia. Pain map overlays for athletes classified with a single entity of groin pain appear to differ visually between clinical entities (Fig. 3). The most frequent areas of pain for each of the clinical entities (marked in orange/red in Fig. 3 and outlined in Fig. 4A) appear to be equivalent to the expected areas of pain for three of the clinical entites; for adductor-related groin pain, the most frequent area of pain corresponds to the proximal adductor longus insertion, for inguinal-related pain, it corresponds to the inguinal canal/inguinal ligament, and for pubic-related groin pain, the pain is central (although appearing slightly cranial to the location of the pubic symphysis). In contrast, the drawings of athletes with iliopsoas-related groin extended medially from the location of the iliopsoas muscle. The pain area outline, where a minimum of two athletes reported their pain, do not appear as distinct, as they show a considerable overlap between the entities, as illustrated in Fig. 4B.

### Pain area, duration and intensity

**Figure 5:**
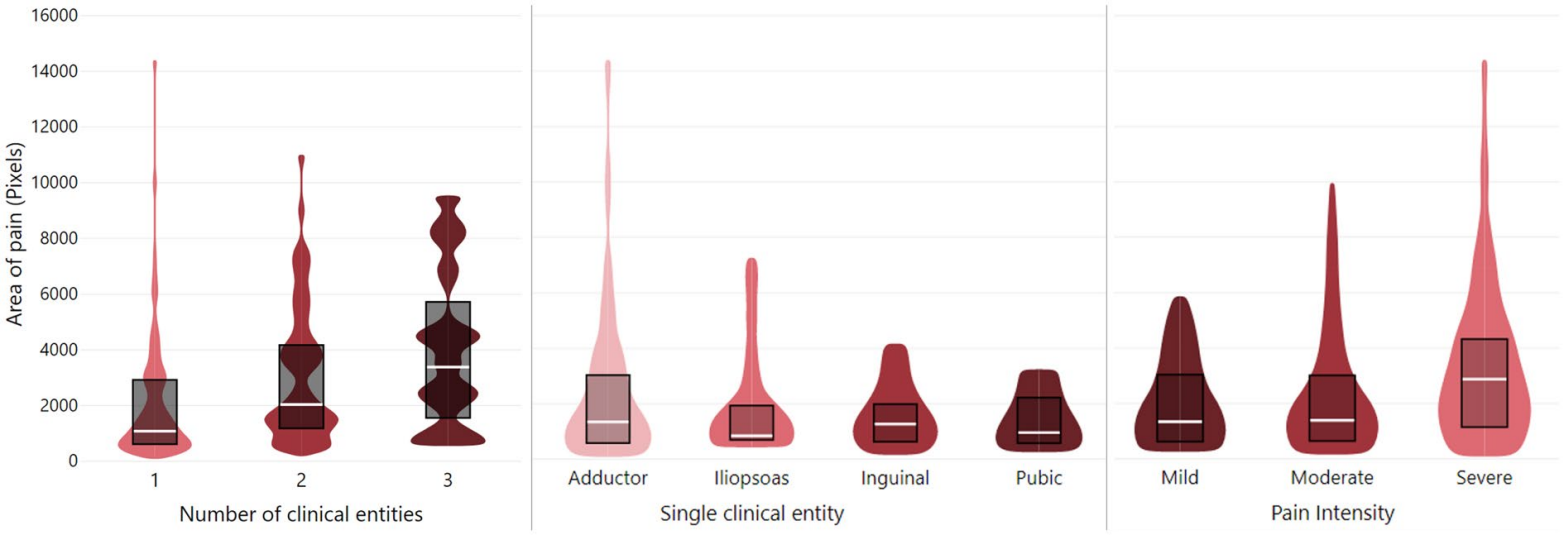
Pain area (expressed in pixels) extracted from digital pain drawings of groin pain, according to (**A**) Number of diagnosed clinical entities: one (N = 88), two (N = 64), and three (N = 15), (**B**) Diagnosed clinical entity in athletes with only one clinical entity: adductor-related (N = 39), iliopsoas-related (N = 18), inguinal-related (N = 24), and pubic-related (N = 7) groin pain, and (**C**) Self-reported intensity of the pain descriptors (mild N = 18, moderate (N = 99), severe (N = 50). White lines signify medians, black lines signify interquartile ranges, and colored area signifies distribution.

Pain descriptor area (pixel count) differed depending on the number of diagnosed clinical entities χ^2^(2) = 16.099, p < 0.001 (Fig. 5A). Post hoc pairwise comparison revealed that pain area differed for athletes diagnosed with one (1198 pixels) clinical entity as compared to two (2189 pixels) (p = 0.006) or three entities (3387 pixels) (p = 0.004), but not between two and three entities (p = 0.734). Independent of the number of diagnosed clinical entities, pain area differed between unilateral (1273 pixels) and bilateral pain (3112 pixels), U = 4902, z = 4.836, p < 0.001. Overall pain areas did not differ between athletes diagnosed with one of the four clinical entities, χ^2^(3) = 0.143, p = 0.986 (Fig. 5B). There was no significant correlation between the duration of groin pain and area for all athletes (Spearman’s rho (ρ) = 0.004, p = 0.958), nor for athletes with a single entity (adductor-related: ρ = 0.012, p = 0.943, inguinal-related: ρ = 0.066, p = 0.771, iliopsoas-related: ρ = 0.364, p = 0.271, pubic-related: ρ = − 0.348, p = 0.499). Pain duration did not differ between athletes with one (72), two (70), and three (90) clinical entities, χ^2^(2) = 2.255, p = 0.324.

The frequency of pain descriptor intensity differed between mild (11%), moderate (59%) and severe (30%), χ^2^(2) = 58.211, p < 0.001. The distributions of pain area significantly differed between mild (75), moderate (79), and severe (103) pain, χ^2^(2) = 8.537, p = 0.014. Post hoc analysis revealed statistically significant differences between the distribution of moderate and severe pain areas (p = 0.018), with patients reporting severe pain having a larger area of pain, assessed by visual inspection of a box plot (Fig. 5C). There was no significant difference in pain intensity between the different clinical entities in patients with a single entity (χ^2^(3) = 0.289, p = 0.962), with mean rank pain intensity scores of 46 for adductor-, 44 for inguinal-, 45 for iliopsoas-, and 48 for pubic-related groin pain. Similarly, pain intensity did not significantly differ between athletes with one (84), two (85), and three (100) clinical entities, χ^2^(2) = 1.882, p = 0.390.

### Pain quality descriptors

**Figure 6:**
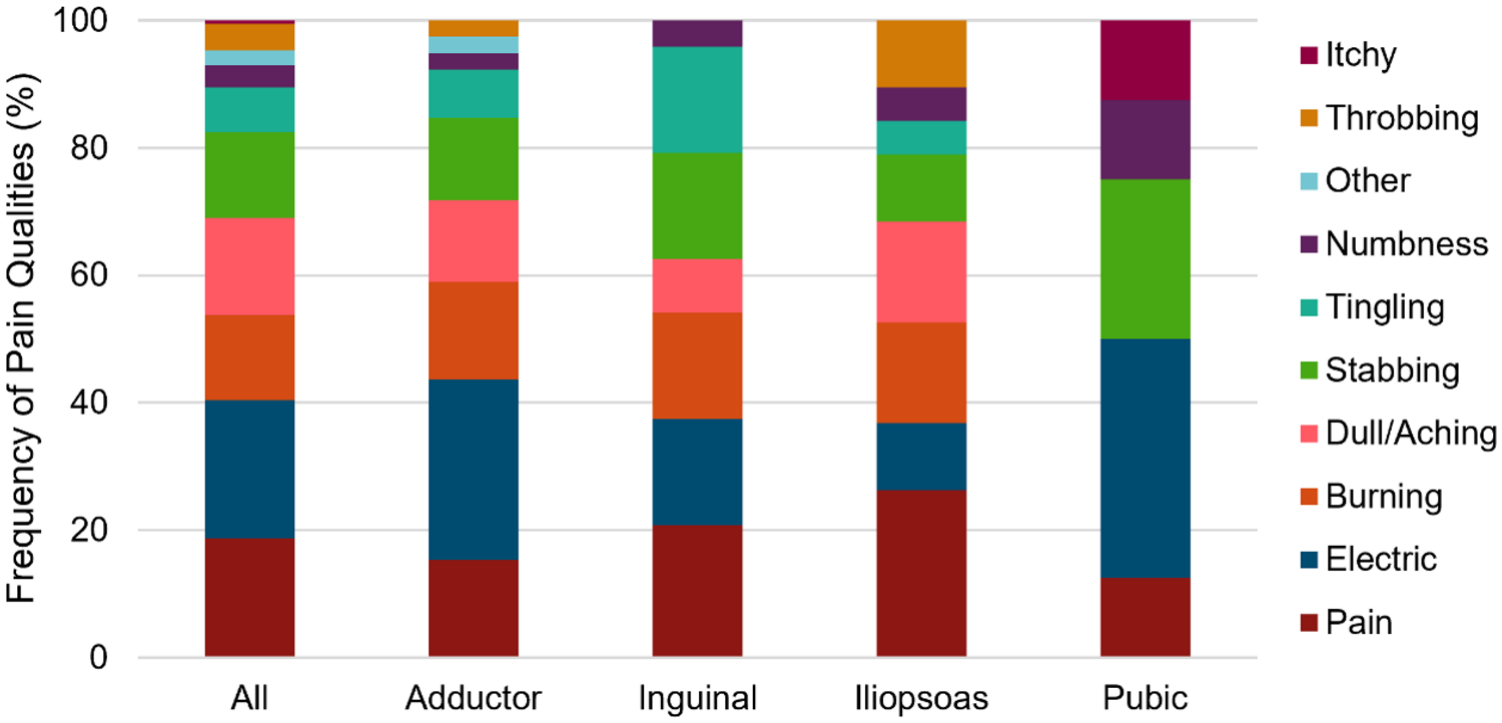
The relative frequency of pain and pain quality descriptors for all athletes (n = 167) and athletes diagnosed with one clinical entity of adductor- (N = 39), inguinal- (N = 24), iliopsoas- (N = 18), or pubic-related (N = 7) groin pain. Color scheme depicts the original color codes utilized by the patients in the drawing platform.

Out of the ten options, the most prevalent pain quality descriptors were electric, pain, and dull/aching, as shown in Fig. 6. Excluding low-frequency descriptors itchy (N = 1) and other (N = 4) in the analysis, the ranked distributions for the area (pixels) of each descriptor were burning (101), dull/aching (90), pain (89), throbbing (87), tingling (84), electric (84), stabbing (62), and numbness (34) differed χ^2^(7) = 14.638, p = 0.041. Post hoc analysis, accounting for multiple comparisons, revealed no statistically significant differences in these distributions (p > 0.187). Excluding the analysis of pubic-related groin pain (N = 7) due to low subject number counts, there was no significant difference in pain descriptor frequency between adductor-, inguinal-, or iliopsoas-related groin (p = 0.893).

## Discussion

We show self-reported pain locations and quality of pain for the four defined clinical entities and one, two or three clinical entities of groin pain in athletes. As shown in the pain map overlay images, visual inspection of the distribution of reported pain and pain qualities differs between the four clinical entities. The most commonly reported pain qualities were electric, dull/aching, and burning and there were no marked differences in these characteristics between the clinical entities. There were no differences in the area (pixel count) associated with each reported pain quality. Nor were there differences in the area between the four clinical entities. Pain area was larger with an increasing number of clinical entities, and bilateral pain also had a greater area than unilateral pain. In this cohort, the area of pain rated as moderate and severe was greater than mild pain.

We present the first study exploring self-reported pain and quality using digital pain drawings in athletes with longstanding groin pain. The pain map overlays for athletes with multiple entities showed widespread pain in the groin area, whereas the overlays for athletes with single entities appeared more distinct. The pain map overlays for athletes with multiple entities appeared as a combination of the pain distribution of the two most frequently diagnosed clinical entities, adductor- and inguinal-related groin pain. These overlay images are likely influenced by the inclusion of athletes, which may represent a different population for clinics with different specialties (e.g. orthopedic surgeons, general surgeons, sports physiotherapists, etc.). Adductor-related groin pain is generally the most common clinical entity in athletes, whereas the prevalence of inguinal-, iliospsoas- and pubic-related groin pain vary more between settings (clinician speciality, athlete type of sport, etc.)^10–12^.

Visual inspection of the overlay images shows that the most frequent pain distributions for athletes classified with a single entity align with the defined clinical entities of groin pain. The results suggest pain location associates with specific regions within the groin area as delineated in the descriptions of each clinical entity, albeit with some overlap. The pain distributions for each clinical entity support the notion that different anatomical structures may drive the pain within the groin area, which can assist in our understanding of the pathology and etiology. Only iliopsoas-related groin pain was not located at the anatomical position of the muscle, but frequently extended medially towards the pubic sympshysis, in line with the inguinal ligament. Additionally, our expectation that some male athletes with inguinal-related groin pain would report pain in the scrotum was unconfirmed. The potential involvement of the genital branch of the genitofemoral nerve can be questioned based on this. The lack of these registrations may however be a methodological limitation if it was a result of the participants failing to select the additional body chart.

The overlapping borders of the pain distributions between each clinical entity for some patients highlight the complexity in individually classifying patients based on self-reported groin pain location. Difficulties arise as pain can be local, diffuse, referred from one or more structures, and can spread beyond the site of an initial injury. Pain location reports become inherently complex when patients present with two or more clinical entities. The most common regions (≈75% of the sub-group populations) show far more differentiation between the single clinical entities. It is important to consider that the accuracy of the self-reported pain location is unknown, and should at this stage only be considered as an assistance to a thorough clinical examination. For situations where clinical examinations are not feasible, such as online consultations or monitoring larger cohorts, further research is needed to examine the accuracy of classifying clinical entities based on individual pain drawings.

As expected, athletes classified with only one clinical entity had a smaller area of pain than athletes classified with multiple entities, whereas there was no difference in pain area between the different single clinical entities. The current clinical opinion is that groin pain starts in one location and can spread or evolve into multiple clinical entities^11,12^. Interestingly, there was no relation between duration and area of pain, and pain duration did not differ between single and multiple entities. These results indicate that the progression or spread of groin pain area with time may be nonlinear or may depend on the location of pain onset. Whether the location of pain onset has predictive value for the development of additional clinical entities remains to be explored. A missed opportunity in this study is a record of pain as being ‘well-defined’ or ‘diffuse.’ A distinction between these two qualitative descriptions may help better understand the accuracy and interpretation of the pain location. Athletes with severe pain had a larger area of pain, indicating pain intensity may be more relevant in the spread of pain than pain duration or location of pain onset.

Assessment of pain qualities in sports injury research is rare. Instead, assessments typically include intensity, onset, and duration. This is the first study to explore pain qualities in groin pain and to contrast characteristics across clinical entities. In clinical practice, assessment of pain quality may be a very important part of clinical reasoning. It may assist the clinician in a more specific diagnosis, including differentiation between nociceptive and neuropathic pain. However in our study, we found no considerable differences in the reported pain qualities between the four defined clinical entities of groin pain.

Approximately 20% of the athletes chose "pain” rather than a specific quality when creating the pain drawing, and pain qualities varied within each clinical entity. Based on these findings reporting a specific pain quality would not facilitate diagnoses according to the Doha agreement meeting classification system. It is unknown whether subclassifcations of pathologies within each of the clinical entities would provide different results. For example, it has been suggested that inguinal-related groin pain has several different possible aetiologies, which can be grouped into neuropathic (peripheral nerve irritation/compression) and nociceptive (musculoskeletal pathologies)^9^. These could generate different pain qualities, with neuropathic pain being associated with electric, burning, and tingling sensations^13^, and nociceptive pain with stabbing, and dull/aching pain^14^. Our results show no clear inclinication towards either neuropathic or nociceptive pain for any of the four clinical entities, but subclassifications may be further explored.

Athletes with multiple entities more frequently had bilateral pain, with less than half of these showing symmetric pain. In comparison, most athletes diagnosed with bilateral adductor-related groin pain had symmetric pain, indicating a uniform underlying pathology within this entity. This is similar to previous reports of pain symmetry in patients with bilateral patellofemoral pain^4^. In contrast, only a few athletes with bilateral inguinal-related groin pain had symmetric pain. This may indicate that there can be different underlying pathologies within this clinical entity for the same individual, although other elements, such as pain severity, may also explain this difference. This study only focused on the classification into clinical entities, and there is currently no gold standard for further diagnostic specification. For future research, individual pain drawings could assist in further exploration of potential subclassifications within the defined clinical entities, and include a focus on other potential causes of groin pain, such as hip-related pain.

We did not include patients with hip-related groin pain or other causes of groin pain due to an expected low prevalence. This means the findings in this study can not be used to distinguish the defined clinical entities of groin pain from other causes of groin pain based on pain location. Similarly, the generalizability to females is limited due to the low number of females included, which also resulted in missing pain map overlays for the females. Additionally, all participants were included from a surgeon’s clinic, and may not represent the pain in athletes who have not reached a level of complaints requiring surgical consideration. Finally, we did not focus on psycho-social factors related to the patients’ pain experiences. The International Association for the Study of Pain emphasises that pain is always subjective, and that psychological factors can influence pain reportings^15^. Further research is required to elucidate whether and how this influences reporting of sports-related groin pain.

## Conclusion

Athletes with longstanding groin pain normally have pain distributions consistent with the areas of the defined clinical entities of adductor-related, inguinal-related, and pubic-related groin. In iliopsoas-related groin pain, the pain extended more medially. This supports the continued use of grouping patient pain according to these clinical entities to differentiate pain locations. On an individual level, there was an overlap between the outlines of the pain drawings reinforcing the challenge in distinguishing specific areas of groin pain according to the Doha agreement classification based on self-reported pain only. The most common pain quality descriptors were electric, pain, and dull/aching, but pain quality was not considerably different between the clinical entities. This means pain quality currently cannot assist in the diagnosis of groin pain according to the clinical entities.

## Data Availability

The datasets used and/or analyzed during the current study available from the corresponding author on reasonable request.
